# A heterozygous variant in the *SLC22A12* gene in a Sri Lanka family associated with mild renal hypouricemia

**DOI:** 10.1186/s12887-018-1185-9

**Published:** 2018-06-29

**Authors:** Dinesha Maduri Vidanapathirana, Subashinie Jayasena, Eresha Jasinge, Blanka Stiburkova

**Affiliations:** 1 Department of Chemical Pathology, Lady Ridgeway Hospital, Colombo, Sri Lanka; 2 Institute of Rheumatology, Prague, Czech Republic; 3 Department of Paediatrics and Adolescent Medicine, First Faculty of Medicine, Charles University and General University Hospital in Prague, Prague, Czech Republic

**Keywords:** Renal hypouricemia, *SLC22A12*, URAT1, Uric acid transporters

## Abstract

**Background:**

Renal hypouricemia is a rare heterogeneous inherited disorder characterized by impaired tubular uric acid transport, reabsorption insufficiency and /or acceleration of secretion. The affected individuals are predisposed to nephrolithiasis and recurrent episodes of exercise-induced acute kidney injury. Type 1 is caused by dysfunctional variants in the *SLC22A12* gene (URAT1), while type 2 is caused by defects in the *SLC2A9* gene (GLUT9). To date, more than 150 patients with the loss-of-function mutations for the *SLC22A12* gene have been found (compound heterozygotes and/or homozygotes), most of whom are Japanese and Koreans.

**Case presentation:**

Herein, we report a nine year old Sri Lankan boy with renal hypouricemia (serum uric acid 97 μmol/L, fractional excretion of uric acid 33%).The sequencing analysis of *SLC22A12* revealed a potentially deleterious missense variant c.1400C > T (p.T467 M, rs200104135) in heterozygous state. This variant has been previously identified in homozygous and/or compound heterozygous state with other causative *SLC22A12* variant c.1245_1253del (p.L415_G417del) in Roma population.

**Conclusions:**

This is the first identification of a family with mild renal hypouricemia1 associated to the p.T467 M variant. Detailed investigations of urate blood and urine concentrations in patients with unexplained hypouricemia are needed and renal hypouricemia should also be considered in patients other than those from Japan and/or Korea. Our finding confirms an uneven geographical and ethnic distribution of Romany prevalent *SLC22A12* variant that need to be considered in Asian patients (population data Genome Aggregation Database: allele frequency in South Asia 0.007055, in East Asia 0.001330).

## Background

Renal hypouricemia (RHUC) is a rare heterogeneous inherited disorder characterized by impaired tubular uric acid transport, reabsorption insufficiency and or acceleration of secretion. RHUC type 1 (OMIM #220150, RHUC1) is characterized by loss-of function mutations (compound heterozygous and/or homozygous) in the *SLC22A12* gene which encodes urate transporter 1 (URAT 1) [1]. Type 2 (OMIM#612076, RHUC2) is caused by the defect in the *SLC2A9* gene which encodes GLUT9 [2]. RHUC is characterized by clinical variability. Although most RHUC1 (about 90%) and RHUC2 patients (< 70%) are asymptomatic detected incidentally, both types may be complicated by nephrolithiasis and acute kidney injury [1–4]. A preliminary diagnosis is based on biochemical markers such as serum uric acid (sUA) concentrations less than 120 μmol/L (reference range: females and children 120–340 μmol/L, males 120–416 μmol/L) and increased fractional excretion of UA (FE-UA, reference range: females 7.3% ± 1.3, males 10.3% ± 4.2) [4, 5]. A final method for diagnosis confirmation and/or identification of the type of RHUC is molecular analysis of the urate transporters *SLC22A12* and *SLC2A9*. An additional method to clarify the effect of novel individual allelic variants of urate transporters in RHUC patients is functional study of urate uptake in *Xenopus* oocyte and HEK cells [6]. No treatment is available; however, patients should be advised to avoid vigorous exercise and to drink plenty of fluids after exercise. Allopurinol has been recently used to prevent recurrence of acute kidney injury [7].

## Case presentation

The proband, 9 year old Sinhalese boy born to unrelated parents after an uncomplicated pregnancy who has an elder sister (14 years) and a younger sister (2 years) presented to our tertiary care children’s hospital in Sri Lanka with abdominal pain and gross haematuria since two weeks. Patient has had repeated episodes of haematuria in the past but urine tests were not available. There was no family history of renal stones. Physical examination was unremarkable. Ultrasonography of abdomen revealed a 2 cm calculus in the right middle moiety of kidney non obstructing. Ultrasound scan of family members was not performed. Biochemical investigations of the proband revealed a persistent hypouricemia (sUA 97 μmol/L, 93 μmol/L). Other biochemical investigations including liver and renal functions were within normal limits. Fractional excretion of uric acid was 33%. Secondary causes of hypouricemia were ruled out. Decreased blood concentrations of UA together with markedly elevated fractional excretion of uric acid (FE-UA) caused us to suspect RHUC and therefore a genetic analysis of the *SLC22A12* and *SLC2A9* gene was performed after informed consent. Probands parents and other two siblings were asymptomatic.

**Fig. 1:**
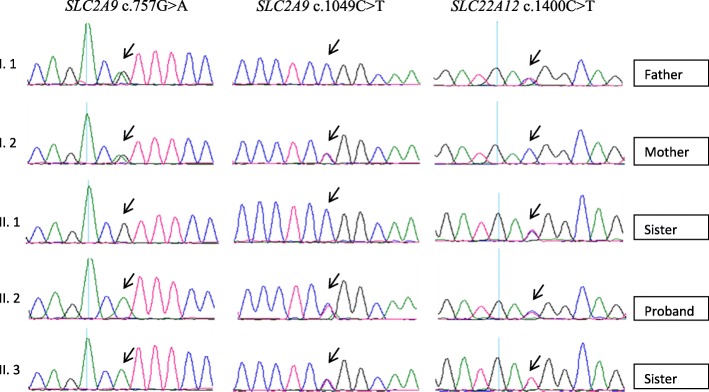
Electropherograms showing part of the *SLC2A9* and *SLC22A12* gene sequence in family with renal hypouricemia*.* The figure show allelic variant p.V282I (c.757G > A) and p.P350L (c.1049C > T) in the *SLC2A9* gene in wild-type (II.1), heterozygote (I.1, I.2) and recessive homozygote state (proband II.2, II.3); and dysfunctional *SLC22A12* variant p.T467 M (c.1400C > T) causes renal hypouricemia in wild-type (I.2) and heterozygous state (I.1, II.1, proband II.2, II.3). Reference sequence: *SLC2A9* NC_000011, region: 64114688..64126396, *SLC22A12* NC_000004, region: 9436946..9650970

The sequencing analysis of *SLC22A12* revealed a previously identified missence variant c.1400C > T (p.T467 M, rs200104135) in heterozygous state [5]. The sequencing analysis of *SLC2A9* revealed two common variants: homozygous c.757G > A (p.V282I, rs16890979) and heterozygous c.962C > T (p.P350L, rs2280205). Analysis of family members identified p.T467 M variant in father of proband (41 year old male: sUA 172 μmol/L, FE-UA 13%) and the two siblings (14 year old sister: sUA 81 μmol/L, FE-UA 15%; two year old sister: sUA 86 μmol/L, FE-UA 25%). The analysis of mother of proband revealed only heterozygous variants p.V2821 and p.P350L in *SLC2A9* (sUA 179 μmol/L, FE-UA 9%). Clinical, biochemical and genetic data are shown in Table 1. Figure 1 shows the electropherograms of part of the SLC2A9 and SLC22A12 gene sequence of the family members.

**Table 1:** Biochemical and Phenotype of proband and family members

	Proband (9 years)	Younger Sister (2 years)	Elder Sister (14 years)	Mother (38 years)	Father (41 years)
Phenotype	Nephrolithiasis‚ Haematuria	Asymptomatic	Asymptomatic	Asymptomatic	Asymptomatic
sUA (μmol/L)	97 (120–320)	86 (120–320)	81 (120–320)	179 (150–350)	172 (150–350)
FE-UA (%)	33 (6–12)	25 (6–12)	15 (6–12)	9 (6–12)	13 (6–12)
Identified variants	*SLC22A12*: c.1400C > T (C/T) *SLC2A9:* c.757G > A (A/A) c.1049C > T (C/T)	*SLC22A12:* c.1400C > T (C/T) *SLC2A9*: c.757G > A (A/A) c.1049C > T (C/T)	*SLC22A12*: c.1400C > T (C/T)	*SLC2A9*: c.757G > A (G/A) c.1049C > T (C/T)	*SLC22A12*: c.1400C > T (C/T) *SLC2A9*: c.757G > A (G/A)

Genomic DNA was extracted from blood sample and/or dry blood spot using a QIAmp DNA Mini Kit (QiagenGmbH, Hilden, Germany) in the Institute of Rheumatology, Prague, Czech Republic. All protein-coding exons of *SLC22A12* and *SLC2A9* were amplified using polymerase chain reaction (PCR) and purified using a PCR DNA Fragments Extraction Kit (Geneaid, Taiwan). DNA sequencing was performed with a DNA sequencer (Applied Biosystems 3130 Genetic Analyzer; Applied Biosystems, USA). Primer sequences and PCR conditions used for amplification were described previously [5, 8, 9]. The reference genomic sequence was defined as version NC_000011.8, region 64,114,688..64126396, NM_144585.3 for *SLC22A12*; NM_020041.2, NP-064425.2,SNP source dbSNP 132 for *SLC2A9*.

## Discussion and conclusions

To date, 41 *SLC22A12* allelic variants and more than 150 compound heterozygous and/or homozygous RHUC1 patients have been found, with the most of the described patients coming from Asia. However, RHUC 1 patients have been described in a variety of ethnic groups (e.g., Arab Israelis, Iraqi Jews, Caucasians, and Roma) and in geographically non-contiguous countries [2, 5, 7, 8, 10, 11]. Moreover, the recent studies found and confirmed the uneven geographical and ethnic distribution of RHUC1 that need to be considered in patients other than those from Japan or the Asia region: the c.1245_1253del and c.1400C > T variants were present in the Roma populations (the largest and the most widespread ethnic minority of Europe with a current European population of 8–10 million) at unexpectedly high frequencies 1.92 and 5.56%, respectively [10–12]. These pathogenic variants frequencies for RHUC are the highest worldwide - to date the high frequency has been reported among Japanese and Koreans: c.774G > A, p.W258X, 2.30–2.37%; c.269G > A, p.R90H, 0.40%; which is indicative of a founder mutation on the Asian continent [13, 14].

Recent data suggest a new concept of renal UA transport: multi molecular complex ‘transportsome’ that probably involves cooperation between multiple urate transporters [15]. The sequencing analysis of *SLC2A9* in family revealed two published common variants: homozygous p.V282I (rs16890979) and heterozygous p.P350L (rs2280205). The potential causal role of the rs16890979 remains unclear: was reported the strongest association with uric acid concentration and gout in women in the Framingham and Rotterdam cohorts [16] and in the island population of the Adriatic coast of Croatia [17]. However, this association did not support the analysis in 541 individuals from Sardinia where rs16890979 was only slightly associated with uric acid concentrations (*p* = 0.02) [18]. Moreover, our previously reported compound approach using association analysis together with functional and immune histochemical characterization of p.V282I and p.P350L variants did not show any influence on expression, subcellular localization, or urate uptake of GLUT9 [9].The analysis of *SLC22A12* revealed the heterozygous dysfunctional variant p.T467 M. The previously reported functional study in *Xenopus* oocyte expression system showed significantly reduced URAT1 signal on the plasma membrane and urate uptake was significantly decreased in comparison with the native protein [5].

Most RHUC patients are clinically asymptomatic and are detected incidently, but some present with nephrolithiasis or haematuria [6]. Our patient presented with nephrolithiasis and haematuria. Biochemical investigations in serum revealed only hypouricemia. Since the proband was confirmed with RHUC the family members were screened. The phenotypic severity of RHUC1 was not correlated with genotype. The genotype c.[1400C > T] was found in the proband, his sister and father. The levels of sUA in proband (97 μmol/L) and siblings (81 and 86 μmol/L) is higher and FE-UA is lower (proband 33%, siblings 15 and 25%) than in other compound heterozygotes and/or homozygotes patients with RHUC1. Although habits and life style were approximately the same in family, the father had sUA and FE-UA within the reference range (172 μmol/L and 13%). These physiological, but marginal levels correspond with a previously described heterozygote for RHUC1 [19]. In addition to sex, several environmental factors are well-known typical risks of elevated of sUA such as obesity, alcohol consumption, and aging. However - the BMI in father is 22.4, alcohol consumption is low, and thus probably effect of sex and age is a significant factor in his increase of sUA.

This fact suggests that one allele of a *SLC22A12* gene contain a variant, c. 1400C > T, while a variant for the other allele is native (e.g. in individuals affected by a recessive disease). Taken together, this leads to the first identification that clinical symptoms of RHUC1 can exists also in heterozygous background of dysfunctional variant of URAT1. Interestingly this variant found in our patient has so far been identified only in the European Roma population (Czech, Slovak and Spanish) population in homozygous and /or heterozygous state [5, 10–12]. Historic, linguistic, and genetic studies identify India as the original homeland of the Roma. Analysis of paternal and maternal lineages as well as autosomal whole-genome studies date the time of the Roma departure from India to approximately 1000 years ago. The data suggest that the group of Roma who left India had a limited size of around 1000 individuals and came from one specific caste or group [20, 21].

Until now, the knowledge of uric acid homeostasis centers its primary investigation on understanding molecular and genetic mechanisms into renal and intestinal uric acid transport. However, a complete understanding of the influence of sUA concentrations for the development and progression of renal diseases and the molecular basis of the pathogenesis of RHUC has not yet been fully elucidated. The previously reported patients with renal hypouricemia and loss-of-function heterozygous variants of URAT1 and/or GLUT9 suggest that also the dominant-negative effect cause RHUC through loss of UA absorption [22]. The function studies of selected URAT1 variants suggested that not only loss-of-function mutation of URAT1 but also the dominant-negative effect cause RHUC through loss of UA absorption, partly due to protein misfolding and accumulation of URAT1 protein in the endoplasmic reticulum [22, 23].

The novelty of the work arises in the founding of heterozygous c.1400C > T variant to be clinically present in family with renal hypouricemia 1 in Sri Lanka. We believe this finding is important for several reasons. Firstly, it highlights the fact that detailed investigations of urate blood and urine concentrations in patients with unexplained hypouricemia are needed. Secondly, the clinical symptoms of RHUC1 can exist also in heterozygous background of URAT1 variant. Thirdly, the identification of p.T467 M variant in Sri Lanka family with renal hypouricemia showed that this causal variant of RHUC1 is not restricted to Roma population as previously thought.

## Abbreviations

DNA: Deoxyribonucleic acid
FE-UA: Fractional excretion of uric acid
HEK cells: Human embryonic kidney
PCR: Polymerase chain reaction
RHUC: Renal hypouricemia
sUA: Serum uric acid
URAT 1: Uric acid transporter 1
